# An extraordinary year

**DOI:** 10.1038/s41467-021-23456-7

**Published:** 2021-05-13

## Abstract

The year 2020 brought unprecedented challenges, and opportunities to reassess and reaffirm our values. As our anniversary year draws to a close, we reflect on achievements and areas for improvement.

At *Nature Communications*, we started 2020 on a positive note in anticipation of our upcoming 10th anniversary in April of that year—then the coronavirus pandemic struck. All of our offices worldwide closed, forcing our editors into lockdown, often repeatedly throughout the year. Working from home they juggled work, care of dependents, lack of suitable workspaces, and mental and physical wellbeing issues.

“We paused to consider how far we had come as a journal in the last 10 years, and where we are heading next, through an Editorial, blog posts and videos.”

We are thankful that we could continue to work from home, unlike many others. We know how difficult the year has been for our authors and reviewers, who faced many challenges while research institutes closed and demands on their time soared. Our thoughts have been particularly with those who have to share their time between research and other, equally important, responsibilities. We urge funders, universities and other stakeholders to take measures to address the conditions under which caregivers (who tend to be disproportionately women) have continued to work in the past year. On our side, we hope that our commitment to disregard from our editorial evaluation any competing works published while a submission to our journal is under review or under revision—and to remain open-minded when submissions overlap with other recently published works—will help relieve some of the pressure our authors feel.

We are most grateful to all of our editors and other team members who worked extra hard to keep the journal running to the best of their abilities in the last year, as well as to our authors and reviewers for their hard-work, patience and adaptability during these unprecedented times.

As our interactions with colleagues, authors and reviewers moved completely online, the initiatives we had planned for our anniversary helped us reflect on our journey as a team. We paused to consider how far we had come as a journal in the last 10 years, and where we are heading next, through an Editorial, blog posts and videos. Our first authors gave us their thoughts on the journey the journal has taken in a Q&A conversation. We asked our authors for their perspectives on how their fields have changed in the past decade and where it was heading in the next ten years in a series of Comments collected here.

The past year has acutely brought to the forefront issues of diversity, equity and inclusion (DEI). Like many other organisations, we embarked on a process of self-reflection and learning to assess how we could reaffirm and strengthen our own support and contributions to DEI in science.

We adhere to the common guiding principles of Nature Portfolio’s commitment to diversity and those laid out by Springer Nature. But we recognise that we have more work to do to educate ourselves and to reach out to under-represented groups of researchers. We have turned inward to examine our editorial practices to enable the thorough and respectful handling of sensitive topics alongside engagement with groups and communities potentially impacted by the research we as a journal publish. We have set up an internal DEI working group currently focusing on activities of outreach as a first concrete step. We are working with diverse communities of researchers to learn about the challenges they face and actions they recommend stakeholders to adopt, and we hope to soon share a series of Comments and Perspectives with our readers.

In a year when we started to focus more on supporting early career researchers (ECRs) through our peer review mentoring programme, we also wished to give those investigators a stronger voice in our journal. We ran a competition for early-career authors asking them to share their research projects and the ups and downs of publishing one of their first papers with us. The four winning entries can be found on our anniversary site here. We started a Q&A series focusing on the challenges ECRs face in their academic lives. In our first conversation, ECRs shared their experiences in navigating virtual conferences and tips for organisers to keep ECRs in mind while planning them. More recently, a Q&A article with three ECRs delved into the issues they are encountering during the pandemic and was accompanied by a conversation with senior investigators and funding representatives sharing their thoughts on how funders and university leadership can better support early career researchers and young faculty.

Our anniversary year has now come to a close, and we look ahead to the next few years: we will continue to support the research community through the publication of important works, and we will keep building on the DEI and ECR initiatives mentioned above in order to do so in the most effective and sensitive way.

